# Analysis of amyloid-like secondary structure in the *Cryab*-R120G knock-in mouse model of hereditary cataracts by two-dimensional infrared spectroscopy

**DOI:** 10.1371/journal.pone.0257098

**Published:** 2021-09-14

**Authors:** Ariel M. Alperstein, Kathleen S. Molnar, Sidney S. Dicke, Kieran M. Farrell, Leah N. Makley, Martin T. Zanni, Usha P. Andley

**Affiliations:** 1 Department of Chemistry, University of Wisconsin, Madison, Wisconsin, United States of America; 2 ViewPoint Therapeutics, San Francisco, California, United States of America; 3 Washington University School of Medicine, Department of Ophthalmology and Visual Sciences St. Louis, St. Louis, Missouri, United States of America; University of Missouri Columbia, UNITED STATES

## Abstract

αB-crystallin is a small heat shock protein that forms a heterooligomeric complex with αA-crystallin in the ocular lens. It is also widely distributed in tissues throughout the body and has been linked with neurodegenerative diseases such as Alzheimer’s, where it is associated with amyloid fibrils. Crystallins can form amorphous aggregates in cataracts as well as more structured amyloid-like fibrils. The arginine 120 to glycine (R120G) mutation in αB-crystallin (*Cryab*-R120G) results in high molecular weight crystallin protein aggregates and loss of the chaperone activity of the protein *in vitro*, and it is associated with human hereditary cataracts and myopathy. Characterizing the amorphous (unstructured) versus the highly ordered (amyloid fibril) nature of crystallin aggregates is important in understanding their role in disease and important to developing pharmacological treatments for cataracts. We investigated protein secondary structure in wild-type (WT) and *Cryab*-R120G knock-in mutant mouse lenses using two-dimensional infrared (2DIR) spectroscopy, which has been used to detect amyloid-like fibrils in human lenses and measure UV radiation-induced changes in porcine lenses. Our goal was to compare the aggregated proteins in this mouse lens model to human lenses and evaluate the protein structural relevance of the *Cryab*-R120G knock-in mouse model to general age-related cataract disease. In the 2DIR spectra, amide I diagonal peak frequencies were red-shifted to smaller wavenumbers in mutant mouse lenses as compared to WT mouse lenses, consistent with an increase in ordered secondary structure. The cross peak frequency and intensity indicated the presence of amyloid in the mutant mouse lenses. While the diagonal and cross peak changes in location and intensity from the 2DIR spectra indicated significant structural differences between the wild type and mutant mouse lenses, these differences were smaller than those found in human lenses; thus, the *Cryab*-R120G knock-in mouse lenses contain less amyloid-like secondary structure than human lenses. The results of the 2DIR spectroscopy study confirm the presence of amyloid-like secondary structure in *Cryab*-R120G knock-in mice with cataracts and support the use of this model to study age-related cataract.

## Introduction

Cataracts affect the majority of people over age 75 and are a major cause of preventable blindness. In addition to the more common age-related cataracts, individuals with mutations in the crystallin gene and other genes develop cataracts at an early age. These early-onset cataracts can be used to study molecular changes that occur with cataract pathogenesis, such as protein structural changes and aggregation. Mammalian α-crystallin is a molecular chaperone, highly expressed in the lens and present at lower levels in other tissues. The two ~20 kDa subunits of α-crystallin, αA and αB, form heterogeneous complexes of 160–1000 kDa. They share 50% sequence identity and are present in a ratio of 3:1 αA:αB in the human lens. α-crystallin is known to aggregate and cause light scattering in aging lenses, and *in vitro*, α-crystallin has been shown to form amyloid fibrils [1]; however, the relative contribution of amyloid-like secondary structure to cataract formation or propagation remains poorly defined [2]. The ability to detect and quantitate amyloid-like fibrils in cataractous lenses would be a major advance in understanding the pathology of cataracts and may aid in the development of drugs that correct protein aggregation and restore lens transparency. Existing techniques to monitor amyloid, such as electron microscopy and Thioflavin T fluorescence [3], are non- or semi-quantitative and of limited sensitivity [1], and so improved techniques to detect and quantify amyloid-like secondary structure in tissue are needed. Additionally, the chaperone system of the lens may sequester small crystallin aggregates, preventing long fibrils from forming and making them difficult to detect by techniques such as electron microscopy [4].

Two-dimensional infrared (2DIR) spectroscopy is an emerging technique that has provided novel structural insights into several proteins from amyloid diseases, including the Aβ oligomers of Alzheimer’s disease [5], the human islet amyloid polypeptide in type 2 diabetes [6], polyQ in Huntington’s disease [7], and the crystallin proteins [8]. The vibrational couplings of the protein amide I backbone carbonyls observed with 2DIR spectroscopy are indicative of protein secondary structures, such as amyloid-like β-sheet, β-sheet, α-helix, and random coil, and secondary structure may be identified by comparison to known standards [5]. *In vitro* 2DIR of αB- and γD-crystallin reveals the highly-ordered β-sheets typical of amyloid-like secondary structure made from crystallins, even when the fibrils are too short or the aggregates are too small to be resolved by transmission electron microscopy (TEM) [9, 10]. Amyloid structures related to Aβ protein were identified in cataractous human lenses of people with Alzheimer’s disease [11]; however, here, we focus on lens tissue that does not contain Aβ protein. Amyloid-like β-sheet structures were first identified in tissue slices of UV-irradiated porcine lenses by their diagonal peak frequency in 2DIR spectra [9]. In *ex vivo* experiments of acid-treated tissues, 2DIR features characteristic of amyloid-like secondary structure were observed, and fibrils >50 nm in length, consistent with amyloid fibrils, were resolved by TEM. In UV-irradiated lens tissues, fibrils were not observed with TEM, but highly ordered β-sheets of amyloid-like secondary structure were identified from the 2DIR spectra [9]. Here, we use the chemistry term *amyloid-like secondary structure* to refer to the highly ordered, vibrationally delocalized secondary structures previously published for lens *Cryab* proteins and human and pig lens tissue after identification with Thioflavin T, 2DIR, or EM [2, 3, 8–10]. *Cryab* proteins *in vitro* demonstrate the characteristic interaction with Congo red indicative of amyloid fibrils [3, 4].

Recent studies using heterodyne 2DIR spectroscopy on postmortem lens tissue from individuals with age-related cataracts showed amyloid-like β-sheet secondary structure and denatured protein structures, supporting the hypothesis that cataracts may be an amyloid disease [10]. UV light induces amyloid-like β-sheet structures, and light scattering increases in regions with amyloid-like structure. No amyloid structures were found in lenses from juveniles, while mature lenses with no cataract diagnosis were also found to contain amyloid-like structures. This data suggests that amyloid-like structures precede opacification, indicating that early detection of amyloid-like secondary structures could be important in identification of cataract disease pathology [10]. Testing for formation of these amyloid-like structures before symptoms appear suggests it may be useful in preventing cataracts [11].

*In vitro* studies suggest that cataracts contain amyloid, but there is little evidence of its existence *in vivo*. In this study, we investigated the amyloid content of lenses from αB-R120G-crystallin mutant (*Cryab*-R120G) knock-in mice using 2DIR spectroscopy. In humans, the αB-R120G-crystallin mutation is associated with early onset congenital cataracts [12]. The *Cryab*-R120G knock-in mice develop cataracts as juveniles, and the crystallins extracted from lenses of these mice show an increase in molecular mass, light scattering, protein aggregation and insolubility [13]. These mice are potentially a useful model for studying cataract-related amyloid formation. Amide I 2DIR spectra have not previously been reported for mouse lenses. The 2DIR spectra of *in situ* lens sections were used to determine the presence of amyloid-like fibrils [10]. *In vitro*, αB-R120G-crystallin forms more amyloid-like fibrils compared to wild-type (WT) αB-crystallin [14]; therefore, we focused on identifying amyloid β-sheet secondary structure in the *Cryab*-R120G knock-in mice. We measured the 2DIR diagonal peak ratios and cross peaks in the WT and mutant lenses and compared these results with previous data on frozen human lenses [10], as well as new data on formalin-fixed, paraffin-embedded (FFPE) human lenses. We demonstrate that the knock-in mice lenses contain some amyloid-like structures, but less than human lenses.

## Materials and methods

### Animals

All animal procedures were approved by the IACUC at Washington University (St. Louis, MO, USA) and conform to the ARVO Statement for the Use of Animals in Ophthalmic and Vision Research. The Mouse Genetics Core at Washington University was responsible for the mouse care, breeding, and genotyping. WT (C57BL/6J) mice (204 days old) and heterozygous knock-in mice carrying the *Cryab*-R120G mutations (194–288 days old) were used. Heterozygous *Cryab*-R120G mutant knock-in mice were previously generated and studied in our laboratory [13, 15]. The knock-in mutant mice were converted to a C57BL/6J background by speed congenics and identified by strain-specific single nucleotide markers (DartMouse, Lebanon, NH, USA). Adult mice were used in these studies. Mice were euthanized by CO_2_ inhalation, consistent with 2013 AVMA guidelines (see S1 Checklist for additional details). Mice were examined by slit lamp biomicroscopy (slit lamp model BG-2GN, serial number 941752; Topcon, Oakland, NJ, USA)] [15]. Prior to observation, the pupils were dilated with a 1% USP tropicamide ophthalmic solution and 10% USP phenylephrine hydrochloride solution in a 9:1 ratio. The slit was placed orthogonal to the mouse for best viewing of the lens opacities. Representative slit lamp images from WT and *Cryab*-R120G heterozygous mice are shown in S1 Fig. No correlation between slit lamp images and 2DIR was investigated due to the small sample size.

### Human lenses

Human lenses were obtained postmortem from the Lion’s Eye Bank of Wisconsin with identifying information removed. The Minimal Risk Institutional Review Board (Health Sciences) at the University of Wisconsin-Madison determined this study was exempt from obtaining informed consent.

### Crystallin proteins

Protein samples were expressed and purified by ViewPoint Therapeutics and shipped to UW-Madison as lyophilized samples. Samples were rehydrated in deuterated 1X PBS (from 10X PBS, Fisher) to a concentration of 3 mg/mL, immediately before use. All 2DIR data was measured at a concentration of 3 mg/mL. Room temperature samples were incubated at room temperature for 27 hours. Heated samples were heated for 2 hours at 43°C and then left at room temperature for 25 hours. TEM samples of crystallin proteins were prepared as previously described [10]. Briefly, equal parts protein (1 mg/mL) and 1% uranyl acetate were pipetted onto a TEM grid immediately before measurement. TEM in Fig 2 were collected with a Tecnai 12 TEM and in S3 Fig, with a Philips CM120 TEM. TIFF images were cropped in Adobe Illustrator to make figures.

### 2DIR measurements

2DIR spectra were collected and processed by methods previously detailed [10]. Briefly, a regenerative amplifier (Spectra Physics, Solstice) was used to pump an optical parametric amplifier (Spectra Physics, TOPAS Prime) to produce signal and idler pulses centered at 1417 and 1845 nm. These pulses were then overlapped at a AgGaS_2_ crystal for difference frequency generation, producing mid IR pulses centered at 6 μm. The mid IR light was split into pump and probe beams. The pump line passed through a Ge acousto-optic modulator pulse shaper to generate the pump pulse pair with a four-frame phase cycling scheme. The pump and probe were overlapped at the sample (with the relative delay between the pump pulse pair and the probe set to maximize the signal strength), after which the probe was directed into a monochromator (Princeton Instruments) and dispersed onto a 64-element mercury cadmium telluride (MCT) array (Infrared Associates). Data was collected using LabView and processed using MATLAB.

Pump pulse delays in time were Fourier transformed into the pump axis in frequency, and probe pulse frequencies were determined by calibrating the MCT array with known water line absorption frequencies, as described previously [10]. The array gives a probe resolution of 2.5 cm^-1^. To compensate for calibration differences from data sets performed in different months, the diagonal slice frequencies were considered to be the same when within the probe resolution (i.e., 1632 = 1633 cm^-1^).

Tissue spectra reported in this report were averaged for 1 minute per location, while crystallin protein spectra were averaged for 25 minutes. All spectra reported were taken with perpendicularly polarized pump and probe beams overlapped at the sample position with a 100 μm diameter focal spot. Spectra are plotted with exponentially spaced contours to emphasize the cross peak. All FFPE lens tissue spectra are normalized to 1641 cm^-1^, while αB-crystallin spectra are normalized to 1639 cm^-1^ and αB-R120G-crystallin spectra are normalized to 1632 cm^-1^.

### Additional methods

The S1 File contains additional methods information for additional sample preparation and information (also see S1 Table), additional 2DIR imaging/data processing information, statistical information, and percent amyloid-like secondary structure estimation.

## Results

### Understanding 2DIR of protein structures

When proteins misfold and assemble into amyloid-like β-sheets, couplings between amide I vibrational modes become large, causing the β-strands to vibrate coherently. The amide I mode is largely due to the protein backbone carbonyls, and these coherent vibrations can therefore provide protein structural information through the diagonal peak frequency, the cross peak location and intensity, and the anharmonicity [8]. Generally, higher frequency, broader peaks in the amide I region correspond to alpha helixes and random coils, due to more localized vibrational modes, while lower frequency, narrower peaks in the amide I region correspond to β-sheet structures due to more delocalized vibrational modes [16].

**Fig 1:**
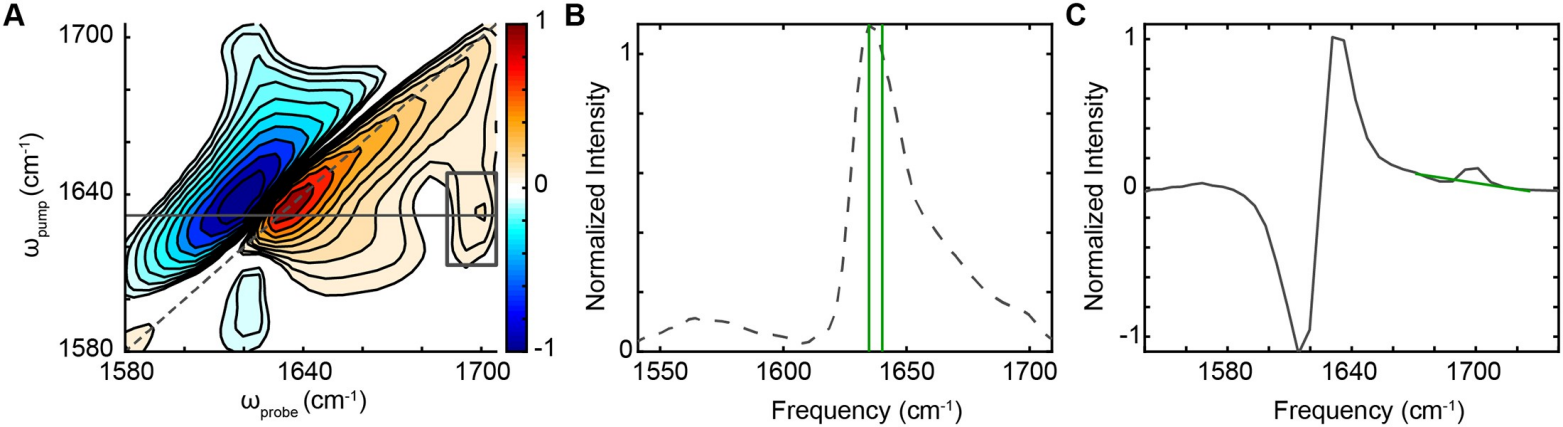
An example of a 2DIR contour plot. (A) A 2DIR contour plot showing data of a 100 μm region of a mutant mouse lens tissue slice. The plot exhibits a pair of positive (red) and negative (blue) peaks that have been normalized on the diagonal at *ω*_pump_ = *ω*_probe_ = 1641 cm^−1^. The plot also exhibits a cross peak (grey box) with a maximum at *ω*_pump_ = 1632 cm^-1^, *ω*_probe_ = 1701 cm^−1^. (B) A plot of the dashed diagonal line in (A) (grey dashed). The vertical lines (green) indicate the frequencies used in determining the diagonal ratio values (1636 and 1641 cm^-1^). (C) A plot of the solid horizontal line in (A) (grey). A baseline (green) is subtracted to give the cross peak intensity. When structures change from native β-sheets to amyloid-like β-sheets, the cross peak intensity increases, and its position shifts to a smaller pump frequency and larger probe frequency.

In 2DIR spectroscopy, a pair of pump pulses excites a vibrational mode (ω_pump_ axis), while a probe pulse measures how system’s modes respond (ω_probe_ axis). An example 2DIR contour plot of one 100 μm diameter region of a *Cryab*-R120G knock-in mouse lens is shown in Fig 1A. The 2D plot shows a red, on-diagonal and a blue off-diagonal peak pair, corresponding to the fundamental and overtone vibrational transitions.

A diagonal slice through a 2DIR spectrum can be interpreted similarly to a Fourier-transform infrared (FTIR) spectrum and these slices are depicted with a positive sign in this work (Fig 1A and 1B, grey dashed). Because the 2DIR signal is proportional to the transition dipole to the fourth power, |μ|^4^, while the FTIR signal is proportional to |μ|^2^, 2DIR spectroscopy can often measure structures that would otherwise be difficult to observe [17]. In particular, amyloid-like β-sheet secondary structures often have μ much larger than native β-sheet secondary structures, and thus can be observed in 2DIR spectra even at lower concentration than native structures present [8, 18].

Additionally, β-sheets display an additional 2DIR spectral signature in cross peaks that indicate coupling between vibrational modes parallel and perpendicular to individual β-strands (Fig 1A, grey box). Here, the cross peak intensity is determined by subtracting a baseline (Fig 1C, green) from the pump slice (Fig 1A and 1C, grey solid line). The cross peak intensity increases, and its position shifts to a smaller pump frequency and larger probe frequency when structures change from native β-sheets to amyloid-like β-sheets. This spectral signature may also be used to indicate the presence of amyloid-like structure, especially in cases where the concentration of amyloid-like structure is so low that changes on the 2DIR diagonal are difficult to distinguish.

### *In vitro* αB-crystallin and αB-R120G-crystallin measurements

**Fig 2:**
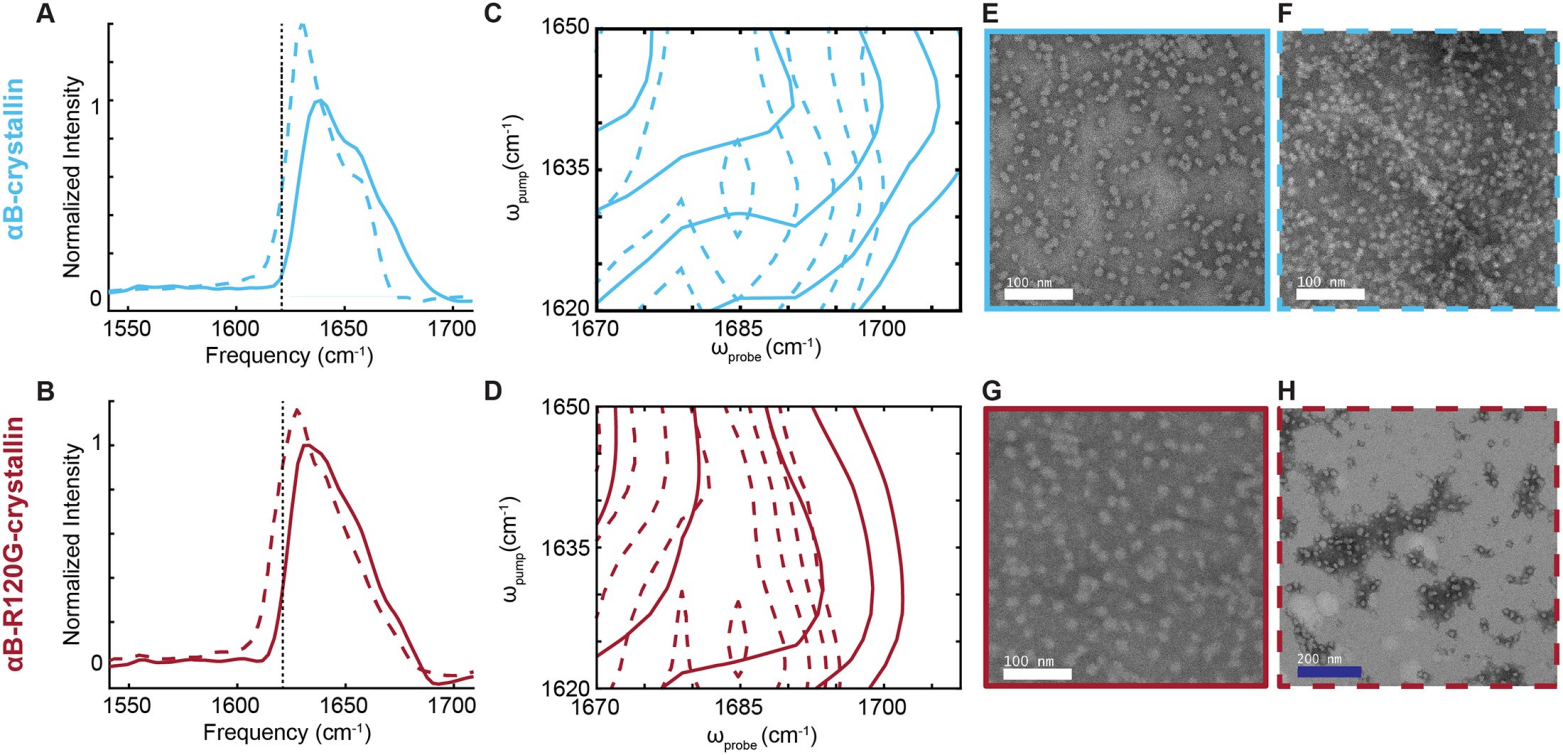
αB-R120G-crystallin has more amyloid-like secondary structure than wild-type αB-crystallin proteins *in vitro* by 2DIR and TEM. (A, B) Overlay of diagonal slices for room temperature αB-crystallin (solid light blue) and heated sample (dashed light blue), or αB-R120G-crystallin (solid maroon) and heated sample (dashed maroon). The dotted vertical line indicates the amyloid-like β-sheet protein secondary structure peak frequency for αB-crystallin, as previously determined through TEM and 2DIR spectroscopy [10]. (C) Overlay of cross peaks for room temperature αB-crystallin (solid light blue) and heated sample (dashed light blue). (D) Overlay of cross peaks for room temperature αB-R120G-crystallin (solid maroon) and heated sample (dashed maroon). (E, F) TEM images for room temperature αB-crystallin (solid light blue) and heated sample (dashed light blue). White scale bars 100 nm. (G, H) TEM images for room temperature αB-R120G-crystallin (solid maroon) and heated sample (dashed maroon). White scale bar at 100 nm, dark blue scale bar at 200 nm. Room temperature samples were incubated at room temperature for 27 hours. Heated samples were heated for 2 hours at 43°C and then left at room temperature for 25 hours.

Prior to comparing the *Cryab*-R120G knock-in mice to the WT mice, *in vitro* samples αB-crystallin and αB-R120G-crystallin were measured. Native β-sheet structures have previously been observed to have diagonal peak frequencies between 1630 and 1640 cm^-1^ [9]. We observed this for both the room temperature αB-crystallin (1639 cm^-1^) and αB-R120G-crystallin samples (1633 cm^-1^) (solid lines, Fig 2A and 2B). When the crystallin samples were heated under mild conditions, their diagonal peak frequencies shifted down to 1631 cm^-1^ and 1626 cm^-1^ (dashed lines Fig 2A and 2B), indicating a slightly more delocalized structure. However, this frequency was not as large of a shift as would have been expected for amyloid-like fibril formation as observed with 2DIR and TEM previously (indicated by black dotted vertical line in Fig 2A and 2B) [9, 10]; 1621 cm^-1^ is a typical frequency expected for well-formed, stable amyloid fibrils. Upon heating under mild conditions, the cross peaks in both samples similarly showed a shift in frequency and increase in intensity that was larger than the native structure but less than that expected for amyloid fibril formation (Fig 2C and 2D, full 2D contour plots shown in S2 Fig). TEM images were taken for all samples (Fig 2E–2H), showing that while typical oligomer structures are observed as expected for the room temperature samples, they are also the dominant feature in the heated samples. The heated αB-crystallin TEM (Fig 2F) shows some larger species similar to those previously observed [9], but neither heated TEM image shows the elongated fibril structures characteristic of mature amyloids. To verify that both proteins can form these structures, samples incubated under acidic conditions were also assessed with 2DIR and show spectral features consistent with amyloid-like β-sheets (S3A and S3B Fig). Acid incubation has previously been used to induce amyloid fibril formation [4], and we observed this effect, as confirmed by the 2DIR features of lower, more intense diagonal peaks in the amyloid-like β-sheet amide I region, as well as a distinct increase in the broad feature of the higher amide I region (1640–1655 cm^-1^) indicating an increase in unfolded protein (S3A and S3B Fig). TEM images of acid-treated αB-crystallin (S3C Fig) did not indicate distinct amyloid structures in the sample, similar to the TEM images of heated protein in Fig 2. Together, this data suggests that the small 2DIR peak frequency shifts and cross peak intensity changes observed reflect the presence of slightly more amyloid-like β-sheet structure in the heated protein samples, while these changes are not apparent by TEM. Similarly, the αB-R120G-crystallin has slightly more delocalized amyloid-like β-sheet structure than the wild-type αB-crystallin protein. 2DIR amyloid-like β-sheet frequencies are inversely proportional to fibril stability [10]. The higher frequencies here suggest that the amyloid fibrils consisting of these crystallins are less stable than other amyloid fibrils and may be more amenable to disaggregation.

### 2DIR images of FFPE WT and *Cryab*-R120G knock-in mouse lens tissue

After initial *in vitro* protein measurements, we measured six *ex vivo* mouse lenses, three from WT mice and three from *Cryab*-R120G knock-in mutants by 2DIR spectroscopy. Thin slices of each FFPE lens were scanned to create 2DIR images.

In our previous work, we determined that frozen juvenile human lens tissue is dominated by native β-sheet structures, with a peak frequency at 1632 cm^-1^, and frozen age-related cataract human lens tissue contained a distribution of native and amyloid-like β-sheet structures with peak frequencies spanning 1615 to 1636 cm^-1^ [10]. In this work, we moved to measuring FFPE tissues, for several reasons. FFPE tissues allowed for better preserved samples that could be imaged over longer periods of time, more precise slices with fewer tears that could be imaged on the setup, and also the ability to establish protocols to inform analysis of tissue types that could only be effectively sectioned when fixed and embedded. However, the new sample environment is quite nonpolar compared to the polar, deuterated environment of the frozen tissue. Thus, the vibrational peak frequencies of interest are shifted in a consistent manner. In order to correct for the tissue preservation method, we conducted a control experiment comparing the 2DIR diagonal peak frequencies for frozen hydrated, dried, and FFPE human lens tissues and observed a shift from native frequencies at 1632 cm^-1^ in frozen tissue up to 1641 cm^-1^ in the FFPE tissue (S4 Fig). For the cataract lens tissue, the peak frequency distribution increased and narrowed as the environment became more nonpolar, so that the dominant feature shifted from 1621 cm^-1^ in the frozen to 1636 cm^-1^ in the FFPE tissue (S4 Fig). The difference in frequency between the native and amyloid-like β-sheet structures therefore decreased from 11 cm^-1^ in frozen to 5 cm^-1^ in FFPE human lens tissue.

An additional control experiment compared frozen and FFPE mouse lens tissue for the WT and *Cryab*-R120G mice (S5 Fig). The same increase in native frequencies up to 1641 cm^-1^ was observed. The frozen knock-in mouse lens tissue was difficult to slice, with many regions or slices rejected for increased tears in the tissue, so a large scale percent of measured locations versus peak frequency graph could not be repeated for the mouse lens as done for the human lens (S4 Fig). The FFPE knock-in mouse lens tissue also had more rips than the WT mouse lens tissue; all ripped tissue regions were rejected from data analysis (see S1 File).

**Fig 3:**
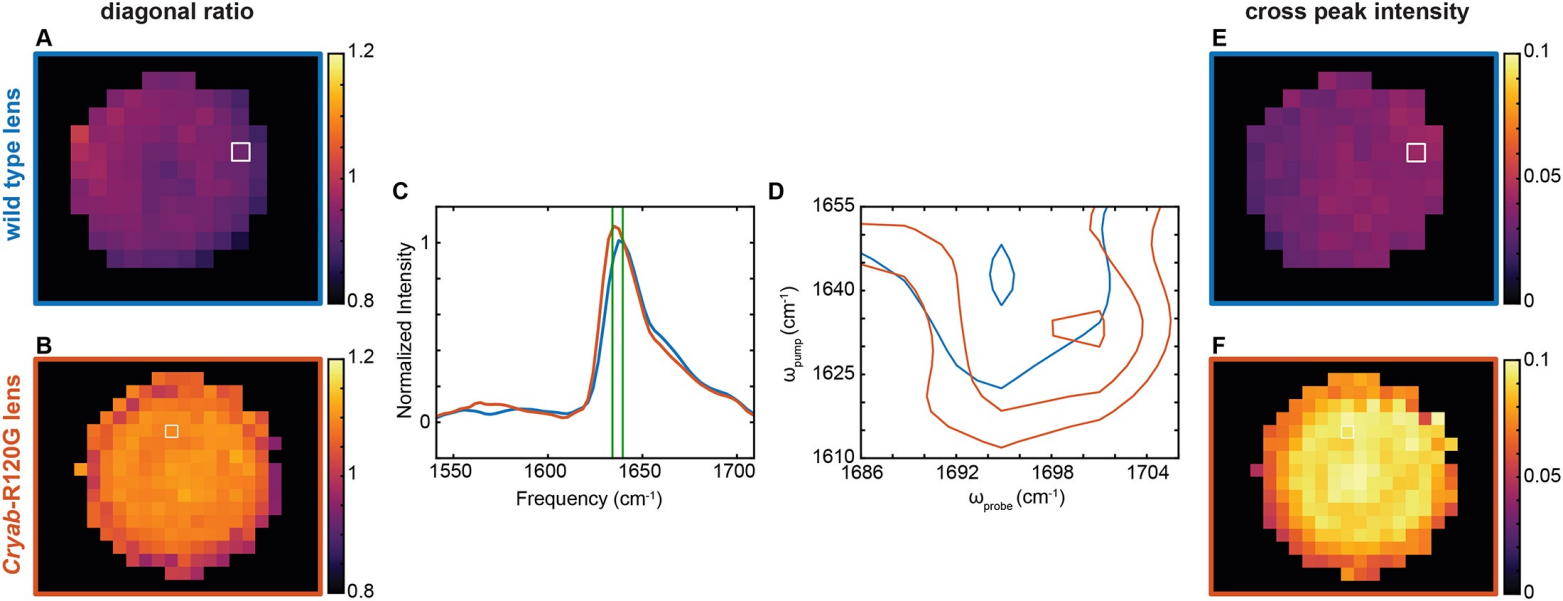
*Cryab*-R120G mutant mouse lenses have more amyloid-like secondary structure features than wild- type mouse lenses by 2DIR image analysis. (A, B) Ratio image of 1636/1641 cm^-1^ intensities for a wild type (A) and mutant (B) mouse lens tissue sections. (C) An overlay of two example diagonal slices for the wild type (blue, from white box in A, E) and *Cryab*-R120G mutant (red, from white box in B, F) mouse lens sections, normalized to 1641 cm^-1^. The vertical lines (green) indicate the frequencies used in determining the diagonal ratio values (1636 and 1641 cm^-1^). (D) An overlay of two example cross peaks for the wild type (blue, from white box in A, E) and *Cryab*-R120G mutant (red, from white box in B, F) mouse lens sections. (E, F) Cross peak intensity image at the *Cryab*-R120G mutant mouse cross peak frequency (ω_pump_ = 1632 cm^-1^, ω_probe_ = 1701 cm^-1^) for the wild type (E) and mutant (F) mouse lens sections.

An example of a WT lens and a *Cryab*-R120G lens 2DIR image is shown in Fig 3A and 3B. To create the 2DIR images, the contour plots are normalized to the native β-sheet peak frequency ω_pump_ = ω_probe_ = 1641 cm^-1^, and the diagonal slice ratio of intensities at 1636 to 1641 cm^-1^ is plotted here. The green lines in Fig 3C indicate where the ratio is taken relative to the entire diagonal slice. These increased ratios for the knock-in mouse lens indicate the presence of more vibrationally delocalized, amyloid-like β-sheet structures. The scale for both the WT lens and the *Cryab*-R120G lens are kept the same, from 0.8 to 1.2, to emphasize the increased ratios for the *Cryab*-R120G lens. An example diagonal slice from the white boxed regions in Fig 3A and 3B is shown in Fig 3C. The *Cryab*-R120G diagonal slice shows an increase in intensity at 1636 cm^-1^. From those same white boxed regions, an example cross peak is shown overlaid in Fig 3D. The *Cryab*-R120G cross peak shows a decrease in pump frequency and an increase in probe frequency to ω_pump_ = 1632 cm^-1^, ω_probe_ = 1701 cm^-1^, as compared to the WT cross peak, indicating a shift to more amyloid β-sheet structures. The cross peak intensities also increase for the *Cryab*-R120G knock-in mouse as compared to the WT lens (Fig 3E and 3F). The scale for both the WT lens and the *Cryab*-R120G lens are kept the same, from 0 to 0.1, again to emphasize the increased values for the *Cryab*-R120G lens. A full set of WT and *Cryab*-R120G knock-in mouse diagonal ratio images, cross peak intensity images, and example 2D contour plots are available in S6 and S7 Figs for all of the lens slices imaged in this work. Overall, these results indicate an increase in amyloid-like secondary structures in the *Cryab*-R120G mutant mouse lens relative to similarly aged wild type controls.

### 2DIR images of FFPE human lenses

**Fig 4:**
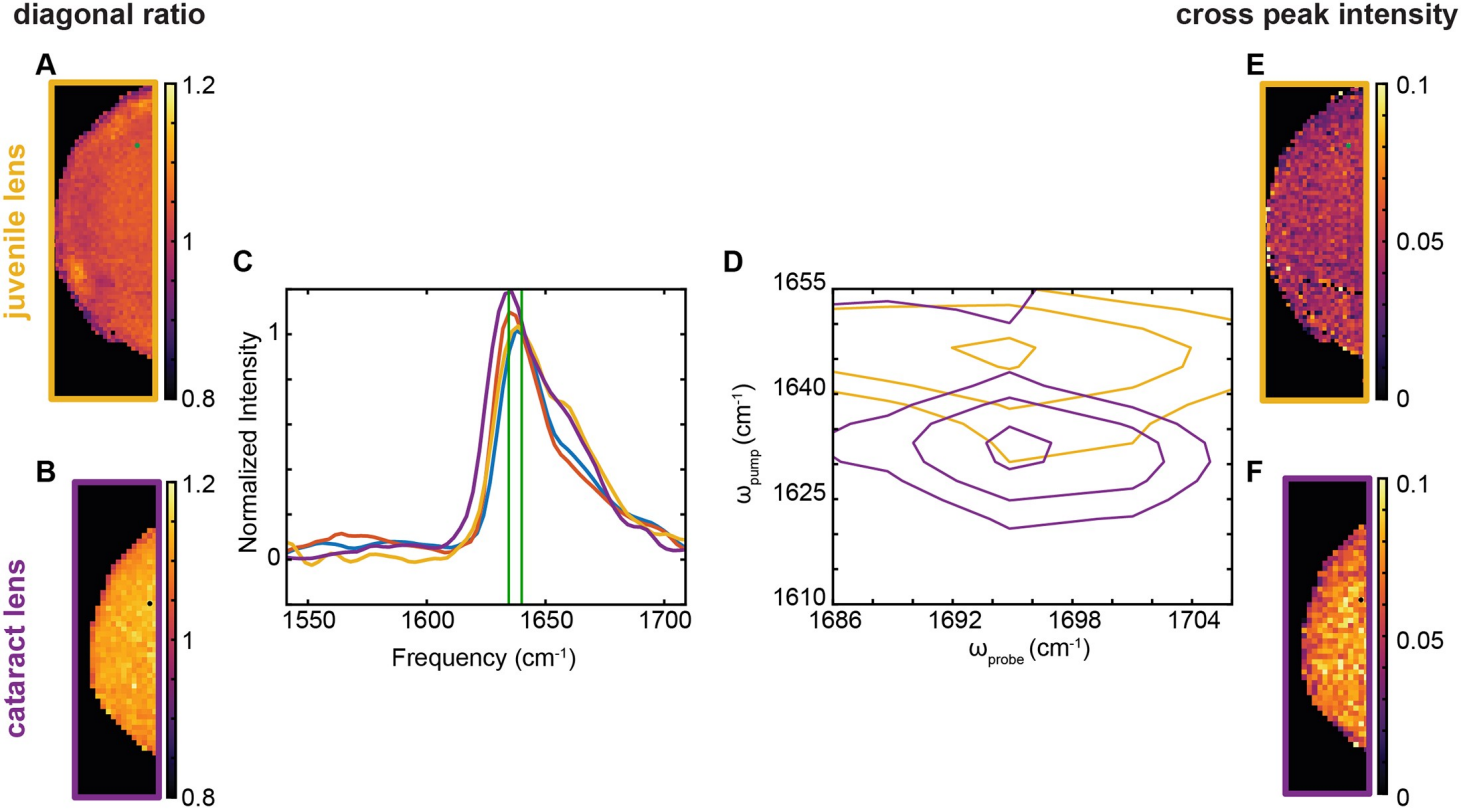
An FFPE age-related cataract human lens tissue contains more amyloid-like secondary structure features than an FFPE juvenile human lens tissue by 2DIR image analysis. (A, B) Ratio image of 1636/1641 cm^-1^ intensities for a juvenile (16 years old, A) and age-related cataract (63 years old, B) human lens tissue sections. (C) An overlay of two example diagonal slices for the juvenile (yellow, from green dot in A, E) and cataract (purple, from black dot in B, F) human lens sections, normalized to 1641 cm^-1^. Additionally, the wild type (blue) and *Cryab*-mutant (red) mouse lens sections from Fig 3 are also shown for comparison. The vertical lines (green) indicate the frequencies used in determining the diagonal ratio values (1636 and 1641 cm^-1^). (D) An overlay of two example cross peaks for the juvenile (yellow, from green dot in A, E) and cataract (purple, from black dot in B, F) human lens sections. (E, F) Cross peak intensity image at the human cataract cross peak frequency (ω_pump_ = 1632 cm^-1^, ω_probe_ = 1695 cm^-1^) for the juvenile (E) and cataract (F) human lens sections.

The diagonal slice ratio and the cross peak intensity features, which are the spectroscopic signatures of amyloid-like β-sheets, have been established through a series of experiments using *in vitro* crystallin proteins and *ex vivo* porcine lens tissue, as well as *ex vivo* human lens tissue [8–10]. To better verify our mouse tissue results and compare them to these previous studies, we wanted to repeat the FFPE measurement for a juvenile human lens and an age-related cataract human lens. A 2DIR ratio image is shown in Fig 4A and 4B. The human lens tissue also shows an increase in the diagonal ratio for the cataract lens as compared to the juvenile lens, indicating more amyloid β-sheet in the cataract lens. An example diagonal slice from the green or black dotted regions in Fig 4A and 4B is shown in Fig 4C. The mice lens diagonal slices from Fig 3C are also overlaid here. The human diagonal slice shows an increased difference between the cataract lens and the juvenile lens as compared to the *Cryab*-R120G knock-in mouse and WT mouse lens samples. The human cataract lens diagonal has a higher intensity at 1636 cm^-1^ than the knock-in mouse lens, indicating more amyloid-like structures in the human cataract than in the knock-in mouse lens. Additionally, both human lens samples have an increased side peak around 1650 cm^-1^, indicating the presence of more α-helix and random coil structures in the human lenses as compared to the mice lenses. From the same green and black dotted regions, an example cross peak is shown overlaid in Fig 4D. The cataract lens cross peak shows a decrease in pump frequency but no change in probe frequency as compared to the juvenile lens (cataract frequency: ω_pump_ = 1632 cm^-1^, ω_probe_ = 1695 cm^-1^). Plotting the cross peak intensity at this new frequency still shows an increase for the cataract lens as compared to the juvenile lens (Fig 4E and 4F). This difference agrees with previous *ex vivo* studies in frozen lens tissue [10]. An example 2D contour plot for the juvenile and cataract human lens is available in S8 Fig. Differences in protein content between the human and mouse lenses might account for the differences in the 1650 cm^-1^ region of the spectrum, and therefore the differences in protein structures between the human and mouse lenses. Overall, these results indicate an increase in amyloid-like structures in the cataract human lens.

We also analyzed the anharmonic shifts for the four lens types measured here (S9 Fig). Anharmonicity is another indicator of protein secondary structure. Larger anharmonicities indicate a localization of the amide I vibrations, which often occurs for denatured proteins with smaller anharmonicity values indicating greater vibrational delocalization, consistent with amyloid-like β-sheet structure [19]. The FFPE human lenses shows a large distribution of anharmonicities, indicating a large range of structures in the lens tissue. The cataract human lens has a smaller median value than the juvenile human lens, indicating amyloid-like β-sheet structure. The mouse lenses have anharmonic shifts that are centered higher than the human lenses, possibly due to their different protein content overall. The mice have much narrower distributions, consistent with the absence of the 1650 cm^-1^ shoulder. The *Cryab*-R120G knock-in mouse has a smaller median anharmonicity value than the WT mouse lens samples, indicating greater vibrational delocalization and more amyloid-like β-sheet structure.

### Estimating the amount of amyloid-like structure

**Fig 5:**
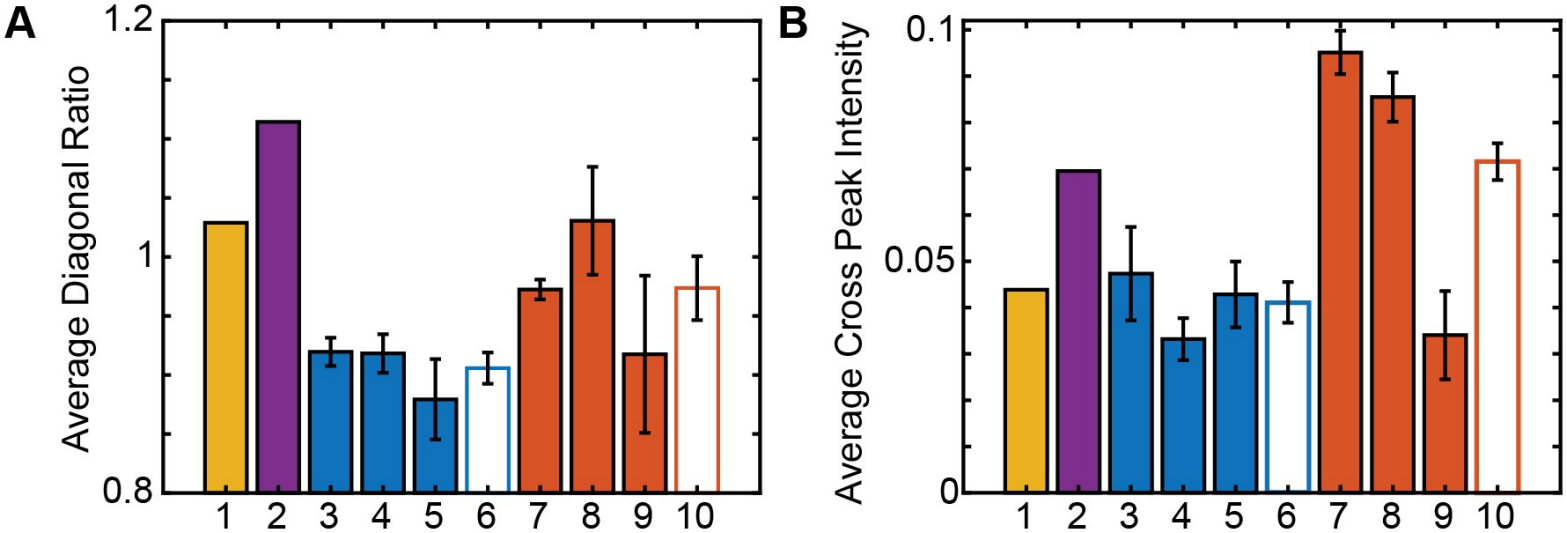
2DIR cross peak intensity reveals significant amyloid-like secondary structure presence in *Cryab*-R120G mouse lenses as compared to wild type lenses. (A) Average diagonal ratios for mouse and human lenses. The cataract lens diagonal ratio is 8.31% larger than the juvenile lens. The *Cryab*-R120G mutant lens diagonal ratio is 7.48% larger than the wild type mouse lens. (B) Average cross peak intensity for mouse and human lenses. The cataract lens cross peak intensity is 58.34% larger than the juvenile lens. The *Cryab*-R120G mutant lens cross peak intensity is 73.91% larger than the wild type mouse lens. Error bars shown are 95% confidence levels. Bars: 1 juvenile human lens, 2 cataract human lens, 3–5 WT mouse lens replicates, 6 average over WT mouse lenses, 7–9 *Cryab*-R120G mutant mouse lens replicates, 10 average over *Cryab*-R120G mutant mouse lenses.

We analyzed the diagonal ratios and cross peak intensities averaged over the individual human and mouse lens slices (Fig 5). The cataract lens diagonal ratio is 8.31% larger than the juvenile lens, and the *Cryab*-R120G lenses diagonal ratio is similarly 7.48% larger than the WT lenses. The cataract cross peak intensities are 58.34% larger than the juvenile human lens, and the *Cryab*-R120G mice cross peak intensities are 73.91% larger than the WT mice lenses. These increases all suggest statistically significant differences between the 2DIR spectra of the mice types, which was verified for a two-tailed t-test at the 95% confidence level (C.L.) for both the diagonal ratio and the cross peak intensity (see S1 File). While slight differences are observed with the diagonal ratio, and these differences are not significant at the 99% or 99.5% C.L., larger differences between the sample types are observed with the cross peak intensity, and these differences are significant at the 99% and 99.5% C.L. (see S1 File). Even when changes on the 2DIR diagonal slice are difficult to distinguish, the cross peak can be used as a structural indicator.

Finding spectral signatures of amyloid-like β-sheet suggests that the *Cryab*-R120G mouse lenses contain these vibrationally delocalized secondary structures (Figs 3 and 4 and S9 Fig). The previously generated *in vitro* crystallin calibration curve was used here to estimate the percent of amyloid-like β-sheet structures in the *Cryab*-R120G mouse lenses [10]. The percent of proteins that adopt amyloid-like β-sheet structures for the *Cryab*-R120G mice is estimated to be between 0 and 3%, smaller than the estimated amyloid-like β-sheet structures for the FFPE human cataract lens shown here (4.08%) (see S1 File). These *Cryab*-R120G mouse lens values are also smaller than the previously calculated percent of amyloid for the frozen human cataract lenses (6.41 ±0.75%), but near the percent of amyloid for the frozen mature human lenses (0–5%) [10]. While the estimate of the percent of amyloid for the *Cryab*-R120G mice is smaller than the percent of amyloid in the human cataract lenses, the spectroscopic signatures of amyloid are still present, therefore the *Cryab*-R120G mice might serve as a good test model to represent human lenses that also contain small amounts of amyloid-like β-sheet protein structures.

## Discussion

UV light induces amyloid-like structures in the lens of porcine tissues [9], and amyloid-like structures are present in human cataract lenses, supporting the hypothesis that cataracts may be an amyloid disease [10]. Previously, in human cataracts, we found that an average of 6.41±0.75% of lens proteins adopted an amyloid-like structure, with some lens regions showing values as high as 40% [10]; about one-third of the locations analyzed in the human lens exhibited mostly denatured structures. In most amyloid diseases, fibrils are identified by TEM, but in cataracts, the fibrils do not appear to be long enough to be obvious.

Small heat shock proteins such as αB-crystallin can prevent amyloid fibril formation by inhibiting both the nucleation and propagation of amyloid fibrils [2]. While αB-crystallin inhibits the further increase in Thioflavin T fluorescence associated with fibril formation of α-synuclein, it cannot disassemble preformed fibrils [20]. αB-crystallin may act by directly inhibiting the supply of partially folded intermediates required for fibril elongation and stable nuclei formation and/or by interacting with the fibril to cap it, thereby preventing further growth [2].

The αB-R120G-crystallin mutation is associated with early onset congenital cataracts in humans [12]. *In vitro*, αB-R120G-crystallin is capable of forming amyloid-like structures, but no fibrils are observed by electron microscopy in lens tissues [2]. αB-R120G-crystallin has decreased chaperone activity *in vitro*; thus, the amyloid-like fibril content in a lens expressing the R120G mutation should increase [21]. Here, we used *ex vivo* 2DIR imaging to determine the presence of amyloid-like fibrils [10] in lenses of WT mice and in *Cryab*-R120G knock-in mice, which serve as a model for hereditary cataracts and myopathy [12, 13], and we compared the results to similar studies in human lenses. The 2DIR spectra revealed higher diagonal peak ratios and higher cross peak intensity for the mutant mouse lenses compared to the WT, indicating that lenses of *Cryab*-R120G knock-in mice contain small amounts of amyloid-like secondary structures. Based on human lens and *in vitro* crystallin measurements, we estimate 0–3% of the knock-in mouse lens protein structure content is amyloid-like β-sheet. These values are consistent with the values for mature lens containing amyloid-like secondary structures formed before clinical diagnosis (0–5%). These results suggest that while the knock-in mouse lenses contain different protein structural content overall from the human lenses, they might serve as a good test model for potential drug-based treatments to prevent or delay the onset of human age-related cataracts.

2DIR can quantify secondary structures through diagonal peak ratios, cross peak intensities, and anharmonicities. Here, the knock-in mice have statistically higher cross peak intensities even when the diagonal peaks cannot be distinguished. Therefore, this spectral signature might be useful in future clinical applications to detect fibril disaggregation. Previously, 2DIR measurements suggested that human lenses have amyloid-like fibrils with frequencies spanning 1615 to 1626 cm^-1^, and these frequencies are inversely proportional to fibril stability [10]. Thus, we expect that positions where the amide I frequency is lower correspond to mature and stable amyloid-like fibrils, whereas at positions with a high amide I frequency, amyloid-like fibrils are less stable. Because the diagonal peak ratios for the *Cryab*-R120G knock-in mice were small, due to higher peak frequencies, the knock-in mice may have less stable amyloid-like β-sheets, and therefore, these amyloid-like β-sheets might be more amenable to disaggregation, and make good test model lenses for disaggregating drug molecules.

Crystallins can form a variety of distinct fibril structures *in vitro* [1]. Chaperones may slow the conversion of these polymorphs, prevent the fibrils from adopting the most stable structures, or prevent their growth. Amyloid fibrils in most diseases form from repeated copies of the same protein, arranged to enable pi-stacking of aromatic side chains, interdigitated or “zippered” side chains, and hydrophobic packing. In contrast, age-related cataracts contain a large mixture of truncated and post-translationally modified crystallins that are the result of oxidation and UVB irradiation, and other heterogenous, non-specific and non-enzymatic modifications [22]. Thus, they may be less likely to form homogeneous fibrils, and if these heterogeneous mixtures of proteins create fibrils we speculate that they will be less stable than other amyloids. We have shown that UV irradiation of αB-crystallin creates peptide fragments, resulting in at least two types of aggregates [9]. Our hypothesis of chaperone-mediated slow fibril maturation and a heterogeneous protein distribution may help explain why long amyloid fibrils are not observed in cataracts. Moreover, our previous studies have shown that an oxysterol molecule can disaggregate amyloid fibrils made of crystallin proteins, while most amyloids are even detergent-insoluble and considered to be an irreversibly aggregated end-state [4, 15, 23]; perhaps this is because the amyloid-like structures present in cataracts are less stable than other amyloids.

Both circular dichroism (CD) and FTIR spectroscopy (the linear form of 2DIR) are commonly used techniques for determining protein structure and are often considered complementary as CD is more historically used for α-helix detection in solution, while FTIR is more suited for β-sheet detection [24]. Due to the signal dependence on the transition dipole, 2DIR can measure amyloid β-sheet secondary structures at even lower concentrations than FTIR, even in the background matrix of native β-sheet structures in tissue [10, 25], necessary for the quantification of the 0–3% amyloid-like structures here. 2DIR measurements can be performed on both the *Cryab*-R120G purified protein and the heterozygous mouse tissue. Early CD measurements demonstrate the difference in first bovine crystallins [26], and then *Cryab* and *Cryab*-R120G structures in solution [21, 27], but more specialized setups are needed for CD measurements of tissue [24], such as CD-SHG [28]. However, with both recent advances in CD for amyloid measurements [29] and the development of related techniques such as chirality induced 2DIR [30], there may be interest in the future for complementary CD investigations.

Our current hypothesis is that amyloid-like structures do contribute to lens opacity in cataracts. Using 2DIR, we have shown that aged and cataractous human lenses contain amyloid, as do lenses from *Cryab*-R120G knock-in mice. Amyloid-like structures formed in the lens may be different from fibrils formed in other diseases; less stable and, therefore, more amenable to disaggregation, perhaps due to the high protein content and the abundance of chaperone proteins in the lens. Thus, it takes longer for lens fibrils to mature into the long stable structures commonly associated with other amyloid diseases. The 2DIR spectroscopy features of young versus old lenses are consistent with a slow progression to more mature amyloid-like fibrils [10]. In these respects, we consider the knock-in mouse *Cryab*-R120G knock-in mice as a useful model for age-related cataracts.

## Supporting information

S1 File Additional methods information. (PDF)

S1 Table Additional lens sample information. (PDF)

S1 Fig Representative slit lamp images of mouse lenses. (A, C) WT and (B, D) *Cryab*-R120G heterozygous mice showing the extent of lens opacity. (A) The WT lens had minor opacities. The *Cryab*-R120G heterozygous lens (B) had increased discrete opacities in nuclear and cortical regions, increased discrete punctate opacities, and overall opacities covering approximately two-thirds of the lens. The *Cryab*-R120G heterozygous lens in (D) had overall opacity. The mice in (A) and (C) were 204 days old, (D) was 205 days old, and (B) was 288 days old. The *small red arrows* indicate reflection from the slit beam. No correlation between slit lamp images and 2DIR was investigated due to the small sample size; both *Cryab*-R120G lenses (S1B and S1D Fig) have overall opacity with high 2DIR cross peak intensity for S1B Fig (Fig 5, bar 8) and low 2DIR cross peak intensity for S1D Fig (Fig 5, bar 9).
(TIF)

S2 Fig 2DIR contour plots for data shown in Fig 2. (A) Contour plot for room temperature αB-crystallin (solid light blue). (B) Contour plot for heated αB-crystallin (dashed light blue). (C) Contour plot for room temperature αB-R120G-crystallin (solid maroon). (D) Contour plot for heated αB-R120G-crystallin (dashed maroon).
(TIF)

S3 Fig A 2DIR and TEM comparison of αB-crystallin and αB-R120G-crystallin with acid treatment. (A) Overlay of diagonal slices for room temperature αB-crystallin (solid light blue, data in Fig 2A), heated sample (dashed light blue, data in Fig 2A), and acid treated sample (dotted light blue). Data has been normalized to 1639 cm^−1^ to match the room temperature sample. (B) Overlay of diagonal slices for room temperature αB-R120G-crystallin (solid maroon, data in Fig 2B), heated sample (dashed maroon, data in Fig 2B), and acid treated sample (dotted maroon). Data has been normalized to 1632 cm^−1^ to match the room temperature sample. (C) TEM image of acid treated αB-crystallin. Dark blue scale bar is 100 nm.
(TIF)

S4 Fig Comparison of frozen, dried, and fixed/paraffin embedded tissue for human lenses. (A) Diagonal slice overlays show a shift to higher frequency and less intensity as the lens tissue goes from the most polar (frozen, rehydrated in buffer) to most nonpolar (paraffin wax) environment. Normalized to juvenile lens tissue peak at 1632 cm^-1^ for frozen, 1636 cm^-1^ for dried, and 1641 cm^-1^ for fixed lens tissue. (B) Peak frequency distributions are heterogeneous, with distinct shifts to higher frequencies as the lens tissue goes from a polar to nonpolar environment. Red: Cataract lens tissue (frozen, then rehydrated in buffer); Blue: Juvenile lens tissue (frozen, then rehydrated in buffer); Purple: Cataract lens tissue (frozen, then dried under nitrogen); Yellow: Juvenile lens tissue (frozen, then dried under nitrogen); Light Blue: Cataract lens tissue (fixed and paraffin embedded); Green: Juvenile lens tissue (fixed and paraffin embedded).
(TIF)

S5 Fig Comparison of frozen and fixed tissue for mouse lenses. (A) Diagonal slice overlays show a shift to higher frequency and less intensity as the lens tissue goes from the most polar (frozen, rehydrated in buffer) to most nonpolar (paraffin wax) environment. Normalized to wild type mouse lens tissue (peak at 1632 cm^-1^ for frozen, 1641 cm^-1^ for fixed). (B) Photographs of fixed, paraffin embedded lens slices shows a lens slice rejected from measurement because of large rips (top left, from *Cryab*-mutant lens sample 3) and a lens slice typical of those used in this study (bottom right, from *Cryab*-mutant lens sample 1). Maroon: *Cryab*-R120G mutant mouse lens tissue (frozen, then rehydrated in buffer); Green: wild type mouse lens tissue (frozen, then rehydrated in buffer); Red: *Cryab*-R120G mutant mouse lens tissue (fixed and paraffin embedded); Blue: wild type mouse lens tissue (fixed and paraffin embedded).
(TIF)

S6 Fig Wild type mouse lens data chart. The wild type mouse lens replicates are listed on the left column, with each slice of tissue measured divided by a horizontal line. The diagonal ratio image, cross peak image, and the 2D contour plot have the same bounds, normalization, and color bars as shown in Fig 3 for the images and Fig 1 for the contour plot. The white dot in the images corresponds to the location of the 2D contour plot shown in the last column. Images that are not a full lens shaped circle were not fully collected (i.e. only half of the lens slice was imaged, or some of the lens slice was ripped off and only the non-ripped portion is imaged). Wild type mouse lens replicate 1 corresponds to bar 3 in Fig 5, wild type mouse lens replicate 2 corresponds to bar 4 in Fig 5, and wild type mouse lens replicate 5 corresponds to bar 5 in Fig 5.
(TIF)

S7 Fig *Cryab*-R120G mouse lens data chart. The *Cryab*-R120G mouse lens replicates are listed on the left column, with each slice of tissue measured divided by a horizontal line. The diagonal ratio image, cross peak image, and the 2D contour plot have the same bounds, normalization, and color bars as shown in Fig 3 for the images and Fig 1 for the contour plot. The white dot in the images corresponds to the location of the 2D contour plot shown in the last column. Images that are not a full lens shaped circle were not fully collected (i.e. only half of the lens slice was imaged, or some of the lens slice was ripped off and only the non-ripped portion is imaged). Mutant mouse lens replicate 1 corresponds to bar 7 in Fig 5, mutant mouse lens replicate 2 corresponds to bar 8 in Fig 5, and mutant mouse lens replicate 3 corresponds to bar 9 in Fig 5.
(TIF)

S8 Fig 2DIR contour plots for data shown in Fig 4. (A) Contour plot for juvenile human lens data (yellow). (B) Contour plot for age-related cataract human lens data (purple).
(TIF)

S9 Fig Comparison of fixed lens anharmonicity values. Percent of locations versus anharmonicity values for juvenile human lens (yellow, top row), cataract human lens (purple, second row), three wild type mouse lenses combined (blue, third row), and three *Cryab*-R120G mutant mouse lenses (red, bottom row).
(TIF)

S1 Checklist Supporting information for animals. (PDF)
