# Supplementary material for: Analysis of amyloid-like secondary structure in the Cryab-R120G knock-in mouse model of hereditary cataracts by two-dimensional infrared spectroscopy
Source: PLoS One. 2021 Sep 14;16(9):e0257098. doi: 10.1371/journal.pone.0257098 (PMC8439473; doi:10.1371/journal.pone.0257098)
Supplement: S1 File — (PDF) [file pone.0257098.s001.pdf]

S1 File. Additional methods information.

Additional sample preparation information

Frozen lens tissue (S4 and S5 Figs) was stored at -20 °C until used. Up to 24 hours before slicing, lenses were removed and refrozen in Tissue Tek OCT medium. Tissues were sliced at -26 °C using a cryo-microtome to a thickness of 25 µm and deposited onto a poly-L-lysine (Electron Microscopy Sciences) treated CaF_2 window (Crystran). Tissues were dried under N2 and then placed along with a 56 µm Teflon spacer into a flow sample cell (Harrick). Deuterated 1X PBS (from 10X PBS, Fisher) was then added before fully sealing the sample cell.

Dried lens tissue was prepared as frozen lens tissue, but no deuterated 1X PBS was added before measurement.

Fixed lens tissue (Figs 1 and 3-5 and S6-S8 Figs) was formalin fixed and paraffin embedded (FFPE processing). Lenses were then sliced to 4 microns thick using a microtome and placed on CaF_2 windows. The slice was then enclosed into a sample cell with a 100 µm Teflon spacer and a second window. No liquid or deuteration was used with fixed samples. Slices were rejected from further measurement if large rips were present, or only unripped portions of the slice were measured if some unripped regions remained.

Acid treated crystallin protein samples were incubated in pD 3 buffer for 27 hours before measurement. The lower pD solution was made by adding DCl to deuterated 1X PBS. As stated in the main text, for crystallin samples, room temperature samples were incubated

at room temperature for 27 hours. Heated samples were heated for 2 hours at 43 °C and then left at room temperature for 25 hours. All samples were prepared at 3 mg/mL protein concentration in deuterated PBS.

2DIR imaging

A motorized translation stage (Thorlabs Kinesis) was used for point map imaging the spectra. The Labview code interfaced between the stage and the detector such that the stage would move and then a 2D spectrum would be collected, repeated until the desired distances were covered. For all lens images in this manuscript, the stage took 100 µm steps, such that each pixel in the images roughly corresponded to the signal of the area of the beam focused at each location. Spectra not measuring the lens slice, such as those of the paraffin wax alone on the CaF_2 window, were rejected by using a lower bound intensity cutoff for the native protein peak in the spectra, which was 0.065 at 1641 cm^{-1} for the data in this paper, where the noise was <0.005 but pump scatter could range up to 0.065 and affect the diagonal peak at the edges of the lens sample.

2DIR data (diagonal peak ratio, cross peak intensity, and anharmonicity)

All data in this report is presented with a positive sign for the fundamental, on diagonal data, depicted as red in the 2D plots, and a negative sign for the overtone, off-diagonal data, depicted as blue in the 2D plots.

Diagonal slices of the 2D spectra were obtained by finding the maximum intensity along the pump axis for each probe axis pixel, using the known water absorptions to calibrate

the pixel frequencies, as discussed in Reference 10. All fixed lens tissue spectra are normalized to 1641 cm^{-1}, while αB-crystallin spectra are normalized to 1639 cm^{-1} and αB-R120G-crystallin spectra are normalized to 1632 cm^{-1}.

The intensity at 1636 cm^{-1} for normalized diagonal slices was used for the diagonal ratio images and bar graphs shown in Figs 3-5 and S6-S7 Figs. Plot scale bars go from a ratio of 0.8 to 1.2.

To create the cross peak images, slices at the constant pump frequency of 1632 cm^{-1} were taken from the normalized 2D spectrum. A baseline from 1671 to 1726 cm^{-1} was subtracted from the data, as shown in Fig 1C. The baseline removes the remaining shoulder of the diagonal peak from the cross peak area. The cross peak intensity at a probe frequency of 1701 cm^{-1} for the mice lenses or 1695 cm^{-1} for the human lenses is then plotted for the cross peak intensity images and bar graph shown in Figs 3-5 and S6-S7 Figs. Plot scale bars go from an intensity of 0 to 0.1.

To determine the anharmonicities for the spectrum, slices at the constant pump frequency of 1632 cm^{-1} were taken from the normalized 2D spectrum. Two Gaussian curves were then fit to the data between 1575 and 1680 cm-1 with the specific lower bounds of [-3 0 0 0 0 0], upper bounds of [0 Inf 12 3 Inf 12], and initial conditions of [-1 1621 6 1 1642 6], where these are given in [a1, b1, c1, a2, b2, c2] format, where a is the peak height, b is the peak center, and c is the peak width for each respective Gaussian. The peak centers of the two Gaussians used for the fit were subtracted and this value was used as the anharmonicity for each spectrum, as plotted in S9 Fig.

Statistical information

Individual values shown in Fig 5A, bars 3-5 and 7-9 were determined by using the MATLAB function mean for the ratios, with the 95% confidence level (C.L.) error bars shown in the figure determined by using the MATLAB function std, and then dividing the result by √N and multiplying that by 1.96. The cross peak intensity bars 3-5 and 7-9 in Fig 5B were similarly determined. At least two slices of each lens were measured, with N slices for each type: N_{WT_bar3}=2, N_{WT_bar4}=4, N_{WT_bar5}=4, N_{mutant_bar7}=4, N_{mutant_bar8}=4, and N_{mutant_bar9}=2.

The data that made up the N slices for each type was comprised of M spectra (data set size after spectra rejected as described above): M_{WT_bar3}=223, M_{WT_bar4}=327, M_{WT_bar5}=262, M_{mutant_bar7}=635, M_{mutant_bar8}=717, and M_{mutant_bar9}=397.

Only 1 slice was used for the juvenile and cataract human lens tissue, so no further evaluations were made. The M spectra comprising these data sets was $M_{juvenile}$=1320 and $M_{cataract}$=420.

A two-tailed t-test was performed to compare wild type mouse lenses with *Cryab*-R120G mutant mouse lenses. First, the average diagonal ratios for the WT and Cryab-R120G mouse lenses were determined by using the MATLAB function mean for the values shown in bars 3-5 or 7-9. Then the error of bars 3-5 or 7-9 was propagated by err=1/3*sqrt(std_a^2 + std_b^2 + std_c^2, where a b and c would be the lens samples for each type, and std_a would be the value shown for the individual error bar. Here, N was considered to be 3, for three lens samples of each type, hence 1/3. The results are shown in Fig 5A, bars 6 and 10 for the diagonal ratios. The cross peak values in Fig 5B bars 6 and 10 were similarly determined.

The two sample t-test performed was

$$t\ statistic = \frac{\bar{x}-\bar{y}}{\sqrt{\frac{s_x^2}{n_x}+\frac{s_y^2}{n_y}}} \qquad (1)$$

where for the diagonal ratios $\bar{x}$=0.974, s_x=0.027, n_x=3, $\bar{y}$=0.906, s_y=0.013, and n_y=3), resulting in a t-statistic of 3.898. For the cross peak intensities, $\bar{x}$=0.072, s_x=0.004, n_x=3, $\bar{y}$=0.041, s_y=0.004, and n_y=3, resulting in a t-statistic of 8.910. Then the t-statistic is used with the MATLAB function tdcf (as in p value =2*(1-tcdf(tstat,df)), where df is 4), the p value is 0.0176 for the diagonal ratios and 0.00088 for the cross peak intensities, resulting in the rejection of the null hypothesis for both the diagonal ratios and the cross peak intensities at a C.L. of 95%. At a C.L. of 99% or 99.5%, the error is calculated using 2.576 or 2.807 instead of 1.96, and the null hypothesis is still rejected for the cross peak intensities (when calculated the same way as above, but with the different multiplier, p_{99}=0.0025, $p_{99.5}$=0.0034), but not for the diagonal ratios. Therefore, the WT and Cryab-R120G mutant mice are significantly different at a C.L. of 95% for both the diagonal ratios and the cross peak intensities. Thus, at 99% or 99.5%, the two mouse lens types are not different when the diagonal ratios are considered, but they are different when the cross peak intensities are considered.

Average percent of amyloid-like secondary structure estimation

Previously, the relationship between the amide I diagonal peak ratio for the spectrum of interest (y) and the percent of amyloid-like β-sheet structure present (x) was determined

to be y=2.0970x+0.875 by using a calibration curve of αB-crystallin and where the intercept was adjusted using frozen human lens tissue [10].

Because this curve was created in a more polar environment than the lens tissue samples, using it to determine the average percent of amyloid-like secondary structure can be considered only an estimate for the mouse samples. If the curve is adjusted for the wild type mouse diagonal ratio average (y=2.0970x+0.906), then the estimated percent amyloid-like β-sheet structure present for the *Cryab*-R120G mutant mouse samples in Fig 5 is 3.22 ± 0.057 %. If the curve is adjusted for the juvenile human lens diagonal ratio average (y=2.0970x+1.029), then the estimated percent amyloid-like β-sheet structure present in the human age-related cataract lens shown in Fig 5 is 4.08%. If the curve is adjusted for the juvenile human lens diagonal ratio average (y=2.0970x+1.029), then the estimated percent amyloid-like β-sheet structure present for the *Cryab*-R120G mutant mouse samples in Fig 5 is -2.64 ± 0.057 %. Therefore the percent amyloid-like β-sheet structure in the *Cryab*-R120G mutant mouse samples is estimated to be between 0 and 3%, smaller than the single fixed human lens sample shown here, and smaller than the frozen human lens samples previously measured (6.4 ± 0.75 %) [10].

Data availability

The data that support the findings of this study are available from the corresponding authors upon reasonable request.
