# Supplementary material for: Analysis of amyloid-like secondary structure in the Cryab-R120G knock-in mouse model of hereditary cataracts by two-dimensional infrared spectroscopy
Source: PLoS One. 2021 Sep 14;16(9):e0257098. doi: 10.1371/journal.pone.0257098 (PMC8439473; doi:10.1371/journal.pone.0257098)
Supplement: S1 Table — (PDF) [file pone.0257098.s002.pdf]

**Sample type**	**Additional information**
Juvenile human lens	16 years old, left eye, female, no ocular history
Age-related cataract human lens	63 years old, right eye, female, ocular history: nuclear sclerosis cataracts, bilateral
Wild type mouse lens (3 in **Fig 5**)	204 days, left eye, female
Wild type mouse lens (4 in **Fig 5**)	204 days, right eye, male
Wild type mouse lens (5 in **Fig 5**)	204 days, right eye, female
Cryab-R120G mouse lens (7 in **Fig 5**)	194 days, left eye, female
Cryab-R120G mouse lens (8 in **Fig 5**)	288 days, left eye, male
Cryab-R120G mouse lens (9 in **Fig 5**)	205 days, left eye, male
