# Supplementary figures and images for: Analysis of amyloid-like secondary structure in the Cryab-R120G knock-in mouse model of hereditary cataracts by two-dimensional infrared spectroscopy
Source: PLoS One. 2021 Sep 14;16(9):e0257098. doi: 10.1371/journal.pone.0257098 (PMC8439473; doi:10.1371/journal.pone.0257098)

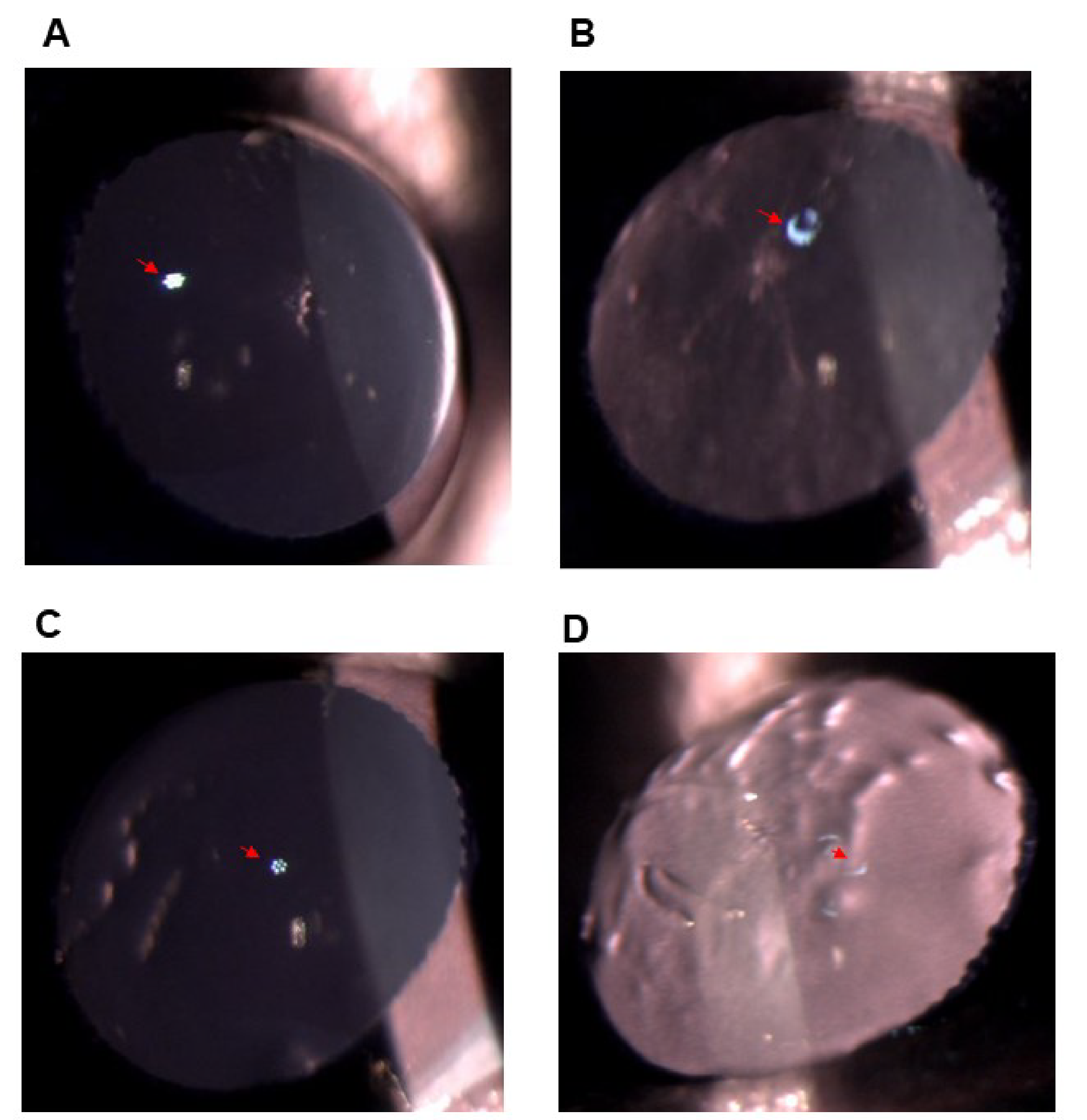

Supplement: S1 Fig — (A, C) WT and (B, D) Cryab-R120G heterozygous mice showing the extent of lens opacity. (A) The WT lens had minor opacities. The Cryab-R120G heterozygous lens (B) had increased discrete opacities in nuclear and cortical regions, increased discrete punctate opacities, and overall opacities covering approximately two-thirds of the lens. The Cryab-R120G heterozygous lens in (D) had overall opacity. The mice in (A) and (C) were 204 days old, (D) was 205 days old, and (B) was 288 days old. The small red arrows indicate reflection from the slit beam. No correlation between slit lamp images and 2DIR was investigated due to the small sample size; both Cryab-R120G lenses (S1B and S1D Fig) have overall opacity with high 2DIR cross peak intensity for S1B Fig (Fig 5, bar 8) and low 2DIR cross peak intensity for S1D Fig (Fig 5, bar 9). (TIF) [file pone.0257098.s003.tif]

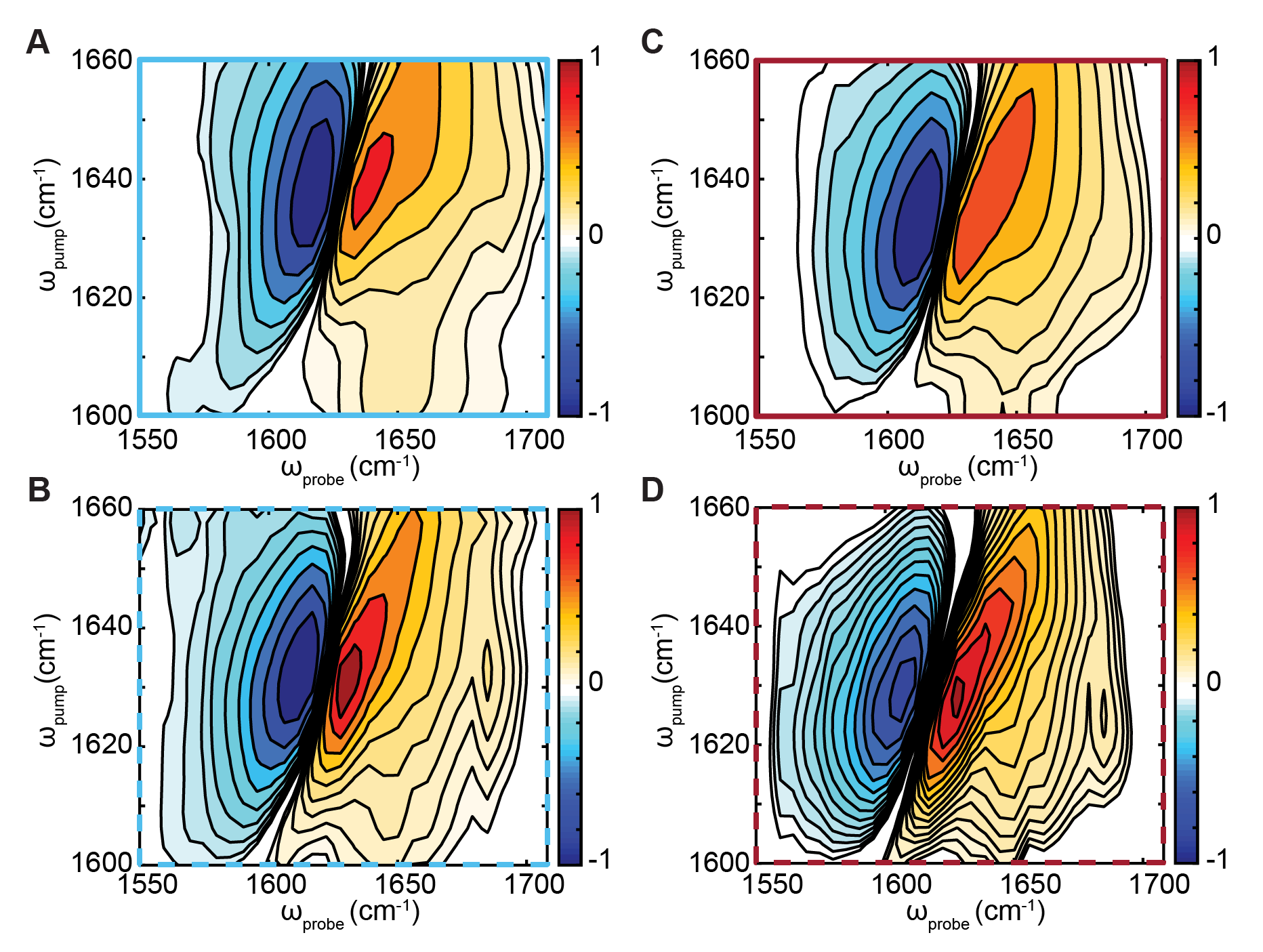

Supplement: S2 Fig — (A) Contour plot for room temperature αB-crystallin (solid light blue). (B) Contour plot for heated αB-crystallin (dashed light blue). (C) Contour plot for room temperature αB-R120G-crystallin (solid maroon). (D) Contour plot for heated αB-R120G-crystallin (dashed maroon). (TIF) [file pone.0257098.s004.tif]

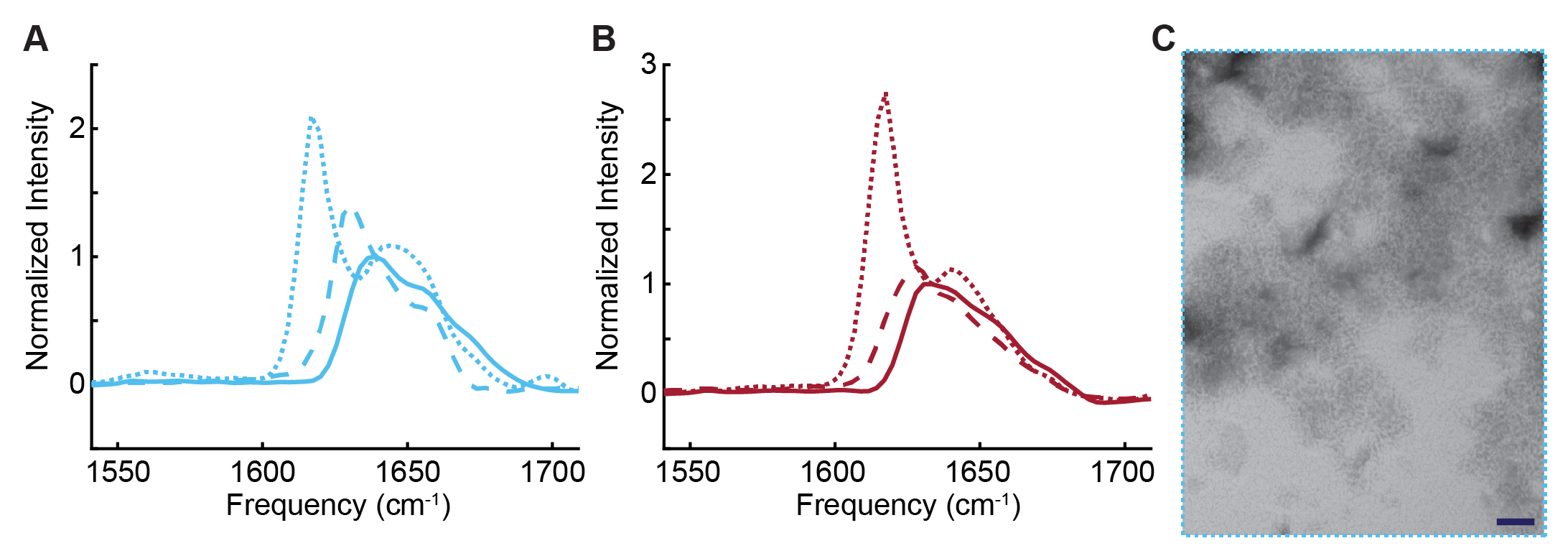

Supplement: S3 Fig — (A) Overlay of diagonal slices for room temperature αB-crystallin (solid light blue, data in Fig 2A), heated sample (dashed light blue, data in Fig 2A), and acid treated sample (dotted light blue). Data has been normalized to 1639 cm−1 to match the room temperature sample. (B) Overlay of diagonal slices for room temperature αB-R120G-crystallin (solid maroon, data in Fig 2B), heated sample (dashed maroon, data in Fig 2B), and acid treated sample (dotted maroon). Data has been normalized to 1632 cm−1 to match the room temperature sample. (C) TEM image of acid treated αB-crystallin. Dark blue scale bar is 100 nm. (TIF) [file pone.0257098.s005.tif]

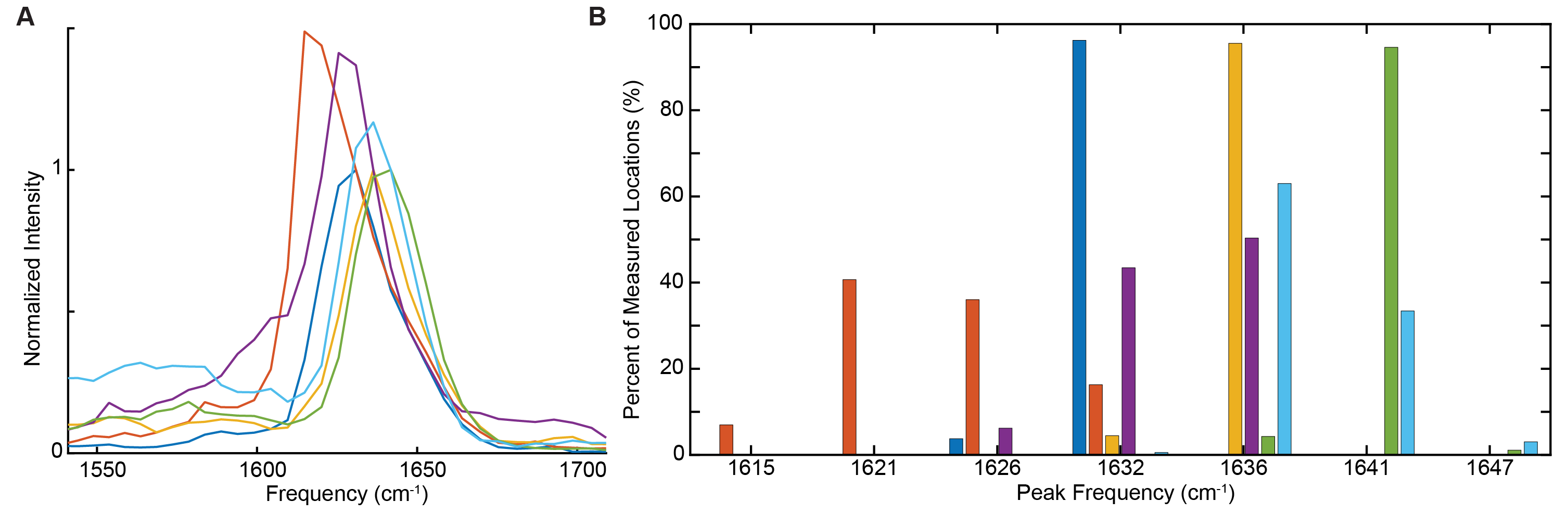

Supplement: S4 Fig — (A) Diagonal slice overlays show a shift to higher frequency and less intensity as the lens tissue goes from the most polar (frozen, rehydrated in buffer) to most nonpolar (paraffin wax) environment. Normalized to juvenile lens tissue peak at 1632 cm-1 for frozen, 1636 cm-1 for dried, and 1641 cm-1 for fixed lens tissue. (B) Peak frequency distributions are heterogeneous, with distinct shifts to higher frequencies as the lens tissue goes from a polar to nonpolar environment. Red: Cataract lens tissue (frozen, then rehydrated in buffer); Blue: Juvenile lens tissue (frozen, then rehydrated in buffer); Purple: Cataract lens tissue (frozen, then dried under nitrogen); Yellow: Juvenile lens tissue (frozen, then dried under nitrogen); Light Blue: Cataract lens tissue (fixed and paraffin embedded); Green: Juvenile lens tissue (fixed and paraffin embedded). (TIF) [file pone.0257098.s006.tif]

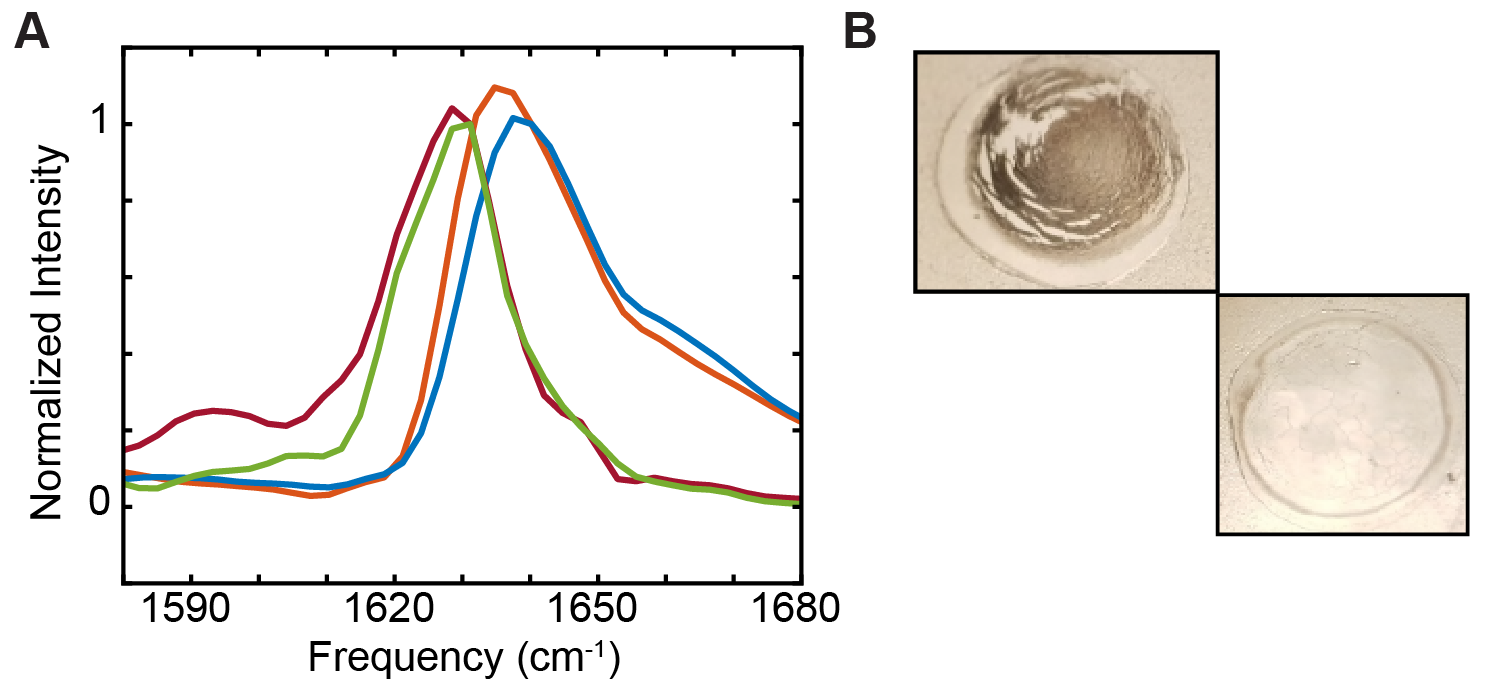

Supplement: S5 Fig — (A) Diagonal slice overlays show a shift to higher frequency and less intensity as the lens tissue goes from the most polar (frozen, rehydrated in buffer) to most nonpolar (paraffin wax) environment. Normalized to wild type mouse lens tissue (peak at 1632 cm-1 for frozen, 1641 cm-1 for fixed). (B) Photographs of fixed, paraffin embedded lens slices shows a lens slice rejected from measurement because of large rips (top left, from Cryab-mutant lens sample 3) and a lens slice typical of those used in this study (bottom right, from Cryab-mutant lens sample 1). Maroon: Cryab-R120G mutant mouse lens tissue (frozen, then rehydrated in buffer); Green: wild type mouse lens tissue (frozen, then rehydrated in buffer); Red: Cryab-R120G mutant mouse lens tissue (fixed and paraffin embedded); Blue: wild type mouse lens tissue (fixed and paraffin embedded). (TIF) [file pone.0257098.s007.tif]

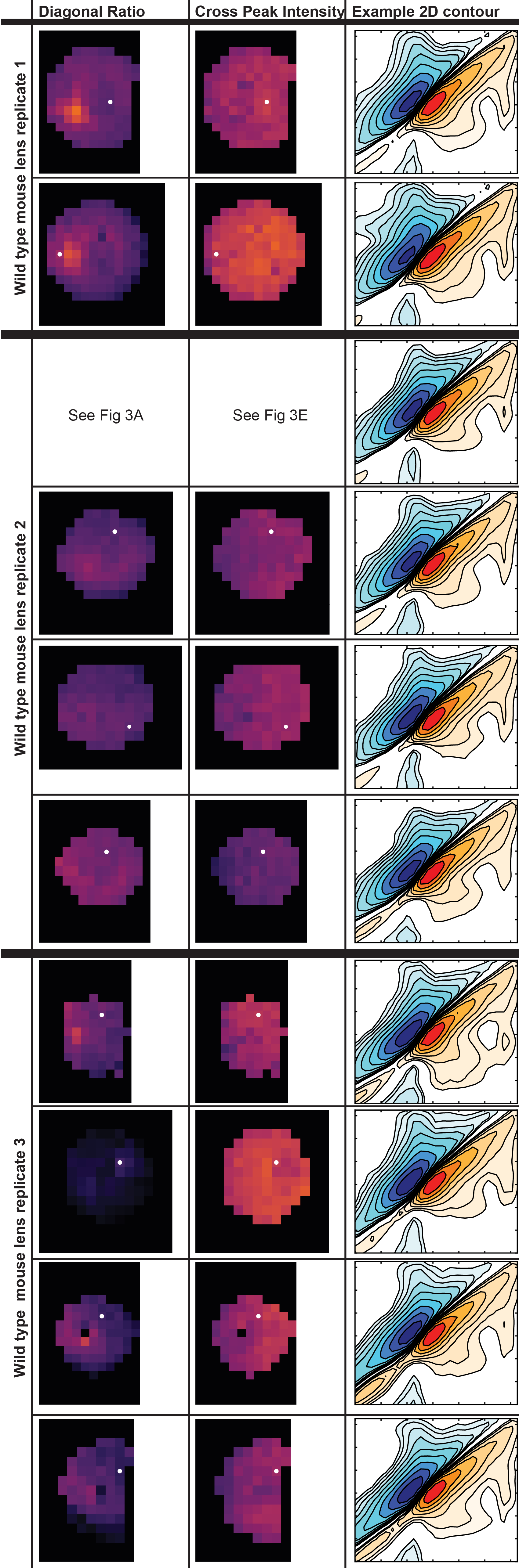

Supplement: S6 Fig — The wild type mouse lens replicates are listed on the left column, with each slice of tissue measured divided by a horizontal line. The diagonal ratio image, cross peak image, and the 2D contour plot have the same bounds, normalization, and color bars as shown in Fig 3 for the images and Fig 1 for the contour plot. The white dot in the images corresponds to the location of the 2D contour plot shown in the last column. Images that are not a full lens shaped circle were not fully collected (i.e. only half of the lens slice was imaged, or some of the lens slice was ripped off and only the non-ripped portion is imaged). Wild type mouse lens replicate 1 corresponds to bar 3 in Fig 5, wild type mouse lens replicate 2 corresponds to bar 4 in Fig 5, and wild type mouse lens replicate 5 corresponds to bar 5 in Fig 5. (TIF) [file pone.0257098.s008.tif]

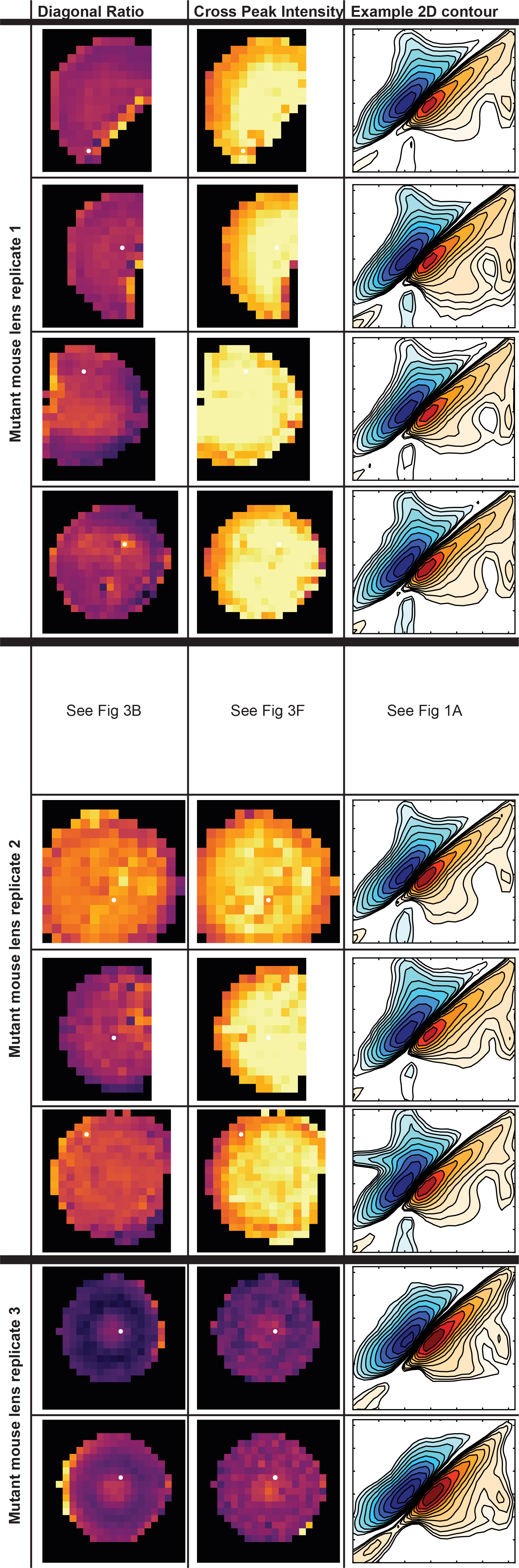

Supplement: S7 Fig — The Cryab-R120G mouse lens replicates are listed on the left column, with each slice of tissue measured divided by a horizontal line. The diagonal ratio image, cross peak image, and the 2D contour plot have the same bounds, normalization, and color bars as shown in Fig 3 for the images and Fig 1 for the contour plot. The white dot in the images corresponds to the location of the 2D contour plot shown in the last column. Images that are not a full lens shaped circle were not fully collected (i.e. only half of the lens slice was imaged, or some of the lens slice was ripped off and only the non-ripped portion is imaged). Mutant mouse lens replicate 1 corresponds to bar 7 in Fig 5, mutant mouse lens replicate 2 corresponds to bar 8 in Fig 5, and mutant mouse lens replicate 3 corresponds to bar 9 in Fig 5. (TIF) [file pone.0257098.s009.tif]

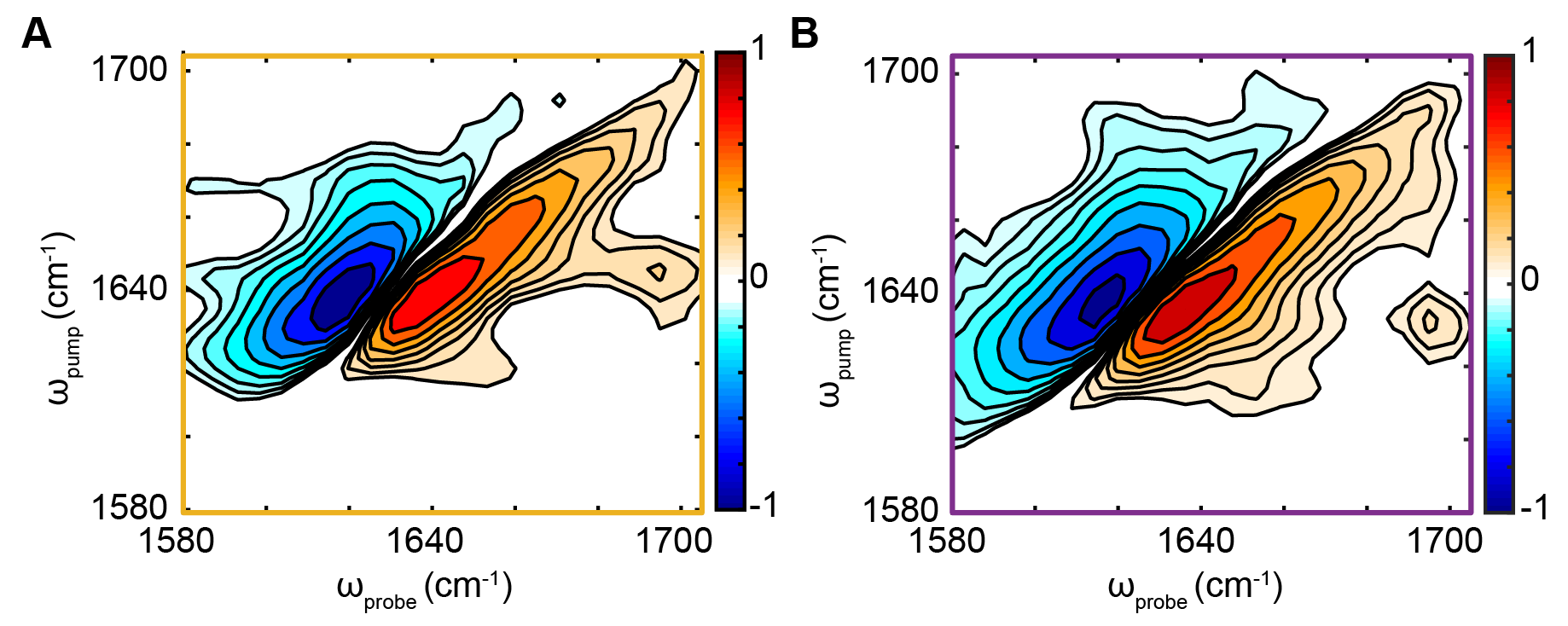

Supplement: S8 Fig — (A) Contour plot for juvenile human lens data (yellow). (B) Contour plot for age-related cataract human lens data (purple). (TIF) [file pone.0257098.s010.tif]

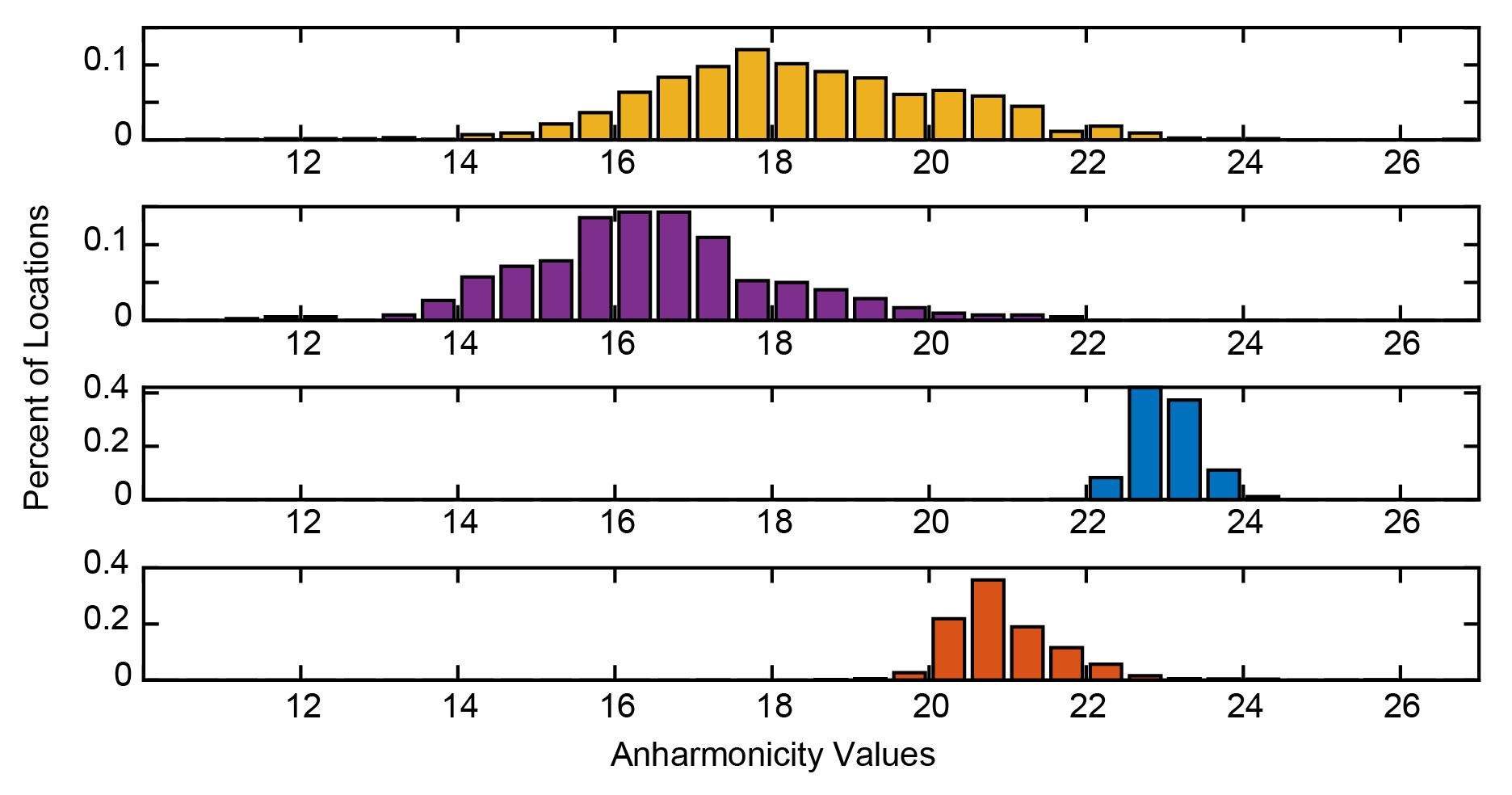

Supplement: S9 Fig — Percent of locations versus anharmonicity values for juvenile human lens (yellow, top row), cataract human lens (purple, second row), three wild type mouse lenses combined (blue, third row), and three Cryab-R120G mutant mouse lenses (red, bottom row). (TIF) [file pone.0257098.s011.tif]
